# Phylogenetic nomenclature and evolution of mannose-binding lectin (*MBL2*) haplotypes

**DOI:** 10.1186/1471-2156-11-38

**Published:** 2010-05-14

**Authors:** Angelica BW Boldt, Iara J Messias-Reason, Diogo Meyer, Carlos G Schrago, Florian Lang, Bertrand Lell, Klaus Dietz, Peter G Kremsner, Maria Luiza Petzl-Erler, Jürgen FJ Kun

**Affiliations:** 1 Institute of Tropical Medicine, University of Tübingen, Tübingen, Germany; 2 Laboratory of Molecular Immunopathology, Hospital de Clínicas, Federal University of Paraná, Curitiba, Brazil; 3 Department of Genetics and Evolutionary Biology, Institute of Bioscience, University of São Paulo, São Paulo, Brazil; 4 Department of Genetics, Federal University of Rio de Janeiro, Rio de Janeiro, Brazil; 5 Institute of Physiology I, University of Tübingen, Germany; 6 Medical Research Unit, Albert Schweitzer Hospital, Lambaréné, Gabon; 7 Department of Medical Biometry, University of Tübingen, Tübingen, Germany; 8 Laboratory of Human Molecular Genetics, Federal University of Paraná, Brazil

## Abstract

**Background:**

Polymorphisms of the mannose-binding lectin gene (*MBL2*) affect the concentration and functional efficiency of the protein. We recently used haplotype-specific sequencing to identify 23 *MBL2 *haplotypes, associated with enhanced susceptibility to several diseases.

**Results:**

In this work, we applied the same method in 288 and 470 chromosomes from Gabonese and European adults, respectively, and found three new haplotypes in the last group. We propose a phylogenetic nomenclature to standardize *MBL2 *studies and found two major phylogenetic branches due to six strongly linked polymorphisms associated with high MBL production. They presented high Fst values and were imbedded in regions with high nucleotide diversity and significant Tajima's D values. Compared to others using small sample sizes and unphased genotypic data, we found differences in haplotyping, frequency estimation, Fu and Li's D* and Fst results.

**Conclusion:**

Using extensive testing for selective neutrality, we confirmed that stochastic evolutionary factors have had a major role in shaping this polymorphic gene worldwide.

## Background

MBL (mannose-binding lectin) is an important component of innate immunity and a central recognition molecule of the lectin pathway of complement, which probably represents the most ancient pathway of complement activation [1]. It binds to an array of carbohydrates such as d-mannose and *N-*acetyl-d-glucosamine on the surface of pathogens and directly opsonizes the microorganism for phagocytosis or activates the complement system via interaction with MBL-associated serine proteases (MASP-1, -2, -3 and Map19). Complement activation kills the pathogen by the membrane-attack complex or by complement-mediated phagocytosis through increased deposition of opsonic C3 fragments. MBL is also able to recognize altered self structures present on apoptotic cells, promoting their clearance, and to modulate the release of various pro-inflammatory cytokines [2,3].

The *MBL2 *genetic polymorphism is responsible for the very common and widespread variation of circulating levels of MBL oligomers and of functional activity of the protein in the human species. This variation is mainly caused by three single nucleotide polymorphisms (SNPs) in the first exon of the gene: MBL2*D (*Arg52Cys*), *B (*Gly54Asp*) and *C (*Gly57Glu*). These mutations have a profound effect on the assembly and stability of the protein, which leads to an increase of low-molecular-mass MBL that has reduced capacity of activating complement and of ligand binding [4,5]. The *D*, *B *and *C *SNPs have been collectively labeled *O*, whereas the major alleles at these loci have been called *A*. The concentration of the protein in serum is further modulated by at least three SNPs in the promoter region: MBL2*H,L (located 550 bp before the transcription start site), *X*, *Y *(located 221 bp before the transcription start site) and *P*, *Q *(non coding SNP located 4 bp after the transcription start site) [6,7]. The combination of structural and promoter polymorphisms results in a dramatic variation in the concentration of high-order MBL oligomers in apparently healthy individuals of up to 1,000-fold (European: range <20-10,000 ng/ml) [8]. Linkage disequilibrium between the SNPs is responsible for only eight haplotypes (as opposed to the 64 theoretically possible) associated with increasingly lower MBL serum concentration: MBL2*HYPA = *LYQA *= *LYPA *>*LXPA *≫ *HYPD *= *LYPB *= *LYQC *= *LYPD *[7,9-13]. Using a haplotyping strategy developed by one of us, we recently defined 14 additional allelic haplotypes, most of them similar to *LYQA *or *LYPA *[2]. Genotypes carrying two copies of either *HYPD*, *LYPB*, *LYQC *or *LYPD *or one of them and *LXPA *are particularly associated with the susceptibility and severity of many diseases, as well as with protection against intracellular infections such as tuberculosis, leprosy and leishmaniasis [14-16].

In this work, we aimed to improve our former analysis by sequencing and haplotyping larger samples of European- and African-derived populations. In order to standardize and simplify comparisons between future association studies, we propose a nomenclature based on the evolutionary convergence of the identified *MBL2 *haplotypes [17]. We tested our samples for the hypothesis of selective neutrality and suggest that stochastic evolutionary factors have had a major role in shaping this polymorphism worldwide.

## Results

To uncover the selective role diseases could have exerted on the *MBL2 *polymorphism, we evaluated the *MBL2 *promoter and exon 1 region from 856 chromosomes of Gabonese adults (this work) and children [2], as well as from 470 chromosomes belonging to individuals of European descent, and compared it with previously published data. Genotype frequencies were at Hardy and Weinberg equilibrium.

**Figure 1:**
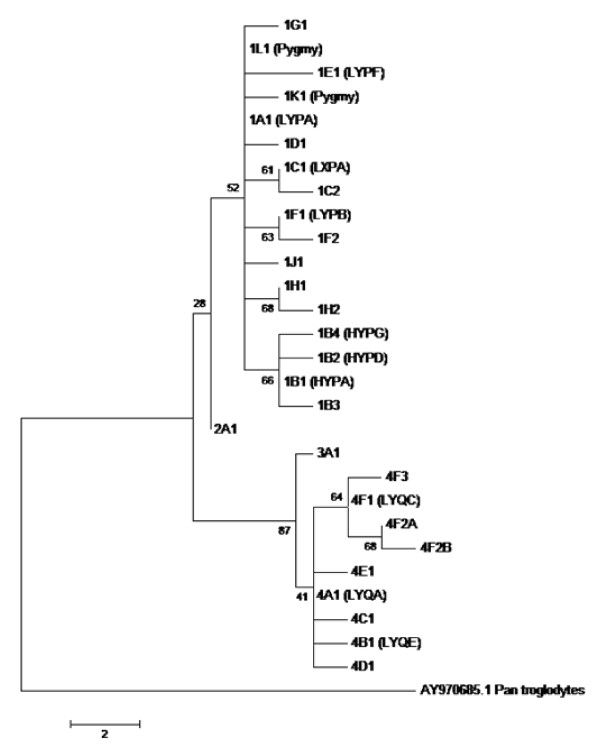
**Maximum parsimony tree with suggested phylogenetic nomenclature (see text)**. The recombinant haplotype *1A1.1B2-l (*LYPD*) was excluded. Bootstrap values are given at nodes of the tree.

*MBL2 *haplotypes identified in this study are listed in Table 1. They were named according to their evolutionary divergence [17] from a hypothetical ancient sequence probably related to *LYQA *and *LYPA *[11,18]. According to the nomenclature system we adopted, the first clades to diverge are numbered with Arabic numerals. The 26 identified haplotypes are divided into two major phylogenetic branches by six polymorphisms (*P1*, *Q1 *or *g.396A *>*C*; *P2*, *Q2 *or *g.474A *>*G*; *P3*, *Q3 *or *g.487A *>*G*; *P4*, *Q4 *or *g.495_500del6*; *P5*, *Q5 *or *g.753C *>*T*, all in strong linkage disequilibrium with the commonly investigated *P6*, *Q6 *polymorphism or *g.826C *>*T*) (Figure 1). Clade *1 is represented by *LYPA *and other haplotypes with *P *variants. Clade *4 is represented by *LYQA *and other haplotypes with *Q *variants. Other clades are represented by the intermediate rare haplotypes previously found by our group in Gabon (*2 *and *3*) [2]. Sublineages of each clade are subsequently designated with capital letters (e.g. *LYQA*-derived haplotypes = *4A and *LYQC*-derived haplotypes = *4F), and individual present-day haplotypes are given Arabic numerals (e.g. *LYQA *= *4A1), following the schema numerals/letters/numerals, if they diverge further (e.g. the *LYQC*-derived haplotype with the *g.797C *>*A *SNP, associated with severe malaria = *4F2A). This system is flexible enough for the accommodation of new haplotypes. For example, we added the *LYPA*-similar haplotypes H16 and H19 found by others exclusively in Pygmy populations [19] as *1K1 and *1L1, and added the *HYPG *haplotype described by us in another study [16], as *1B4*. It is however not suited for recombinant haplotypes. In this case, we chose to call them by the names of the parental haplotypes, separated by a dot. *LYPD *for example is most probably the product of a recent intragenic recombination event between *HYPD *(*1B2) and *LYPA *(*1A1) or *LYPB *(*1F1) [20]. Since the recombination between *HYPD *and *LYPB *would have generated *HYPB*, which has not been found, we arbitrarily chose to call this haplotype *1A1.1B2 (equivalent to *LYPA *× *HYPD*). We also wished to incorporate reported associations of haplotypes with MBL concentration. In order to do this, we added a dash followed by small capitalized "h" or "l" letters, referring to "high" or "low" MBL levels in serum, respectively (e.g. *LYQA *= *4A1-h).

**Table 1:** Nucleotide changes and haploypes of *MBL2*.

	259	273	311	388	396	456	474	477	478	482	487	495	578	598	602	658	659	712	753	788	797	826	925	926	965	1045	1052	1061
		(*H*, *L*)			(*P1*, *Q1*)		(*P2*, *Q2*)				(*P3*, *Q3*)	(*P4*, *Q4*)			(*X*, *Y*)				(*P5*, *Q5*)			(*P6*, *Q6*)				(*A*, *D*)	(*A*, *B*)	(*A*, *C*)
*1A1-h (*LYPA*)	C	C	G	G	A	G	A	C	G	A	A	AAAGAG	G	C	G	C	C	A	C	T	C	C	C	T	G	C	G	G
*1A1.1B2-l (*LYPD*)	.	.	.	.	.	.	.	.	.	.	.	.	.	.	.	.	.	.	.	.	.	.	.	.	.	T	.	.
*1B1-h (*HYPA*)	.	G	.	.	.	.	.	.	.	.	.	.	.	.	.	.	.	.	.	.	.	.	.	.	.	.	.	.
*1B2-l (*HYPD*)	.	G	.	.	.	.	.	.	.	.	.	.	.	.	.	.	.	.	.	.	.	.	.	.	.	T	.	.
*1B3	.	G	.	.	.	.	.	.	.	.	.	.	.	A	.	.	.	.	.	.	.	.	.	.	.	.	.	.
*1C1-l (*LXPA*)	.	.	.	.	.	.	.	.	.	.	.	.	.	.	C	.	.	.	.	.	.	.	.	.	.	.	.	.
*1C2	.	.	C	.	.	.	.	.	.	.	.	.	.	.	C	.	.	.	.	.	.	.	.	.	.	.	.	.
*1D1-h	T	.	.	.	.	.	.	.	.	.	.	.	.	.	.	.	.	.	.	.	.	.	.	.	.	.	.	.
*1E1-h (*LYPF*)	.	.	.	.	.	.	.	.	.	.	.	.	.	.	.	.	.	.	.	.	.	.	G	G	.	.	.	.
*1F1-l (*LYPB*)	.	.	.	.	.	.	.	.	.	.	.	.	.	.	.	.	.	.	.	.	.	.	.	.	.	.	A	.
*1F2	.	.	.	.	.	.	.	.	.	.	.	.	.	.	.	.	.	.	.	C	.	.	.	.	.	.	A	.
*1G1-h	.	.	.	.	.	.	.	.	.	.	.	.	.	.	.	A	.	.	.	.	.	.	.	.	.	.	.	.
*1H1-h	.	.	.	A	.	.	.	.	.	.	.	.	.	.	.	.	.	.	.	.	.	.	.	.	.	.	.	.
*1H2-h	.	.	.	A	.	.	.	T	.	.	.	.	.	.	.	.	.	.	.	.	.	.	.	.	.	.	.	.
*1J1-h	.	.	.	.	.	.	.	.	A	.	.	.	.	.	.	.	.	.	.	.	.	.	.	.	.	.	.	.
*2A1-h	.	.	.	.	.	.	.	.	.	.	G	.	.	.	.	.	.	.	.	.	.	.	.	.	.	.	.	.
*3A1-h	.	.	.	.	.	.	G	.	.	.	G	------	.	.	.	.	.	.	T	.	.	T	.	.	.	.	.	.
*4A1-h (*LYQA*)	.	.	.	.	C	.	G	.	.	.	G	------	.	.	.	.	.	.	T	.	.	T	.	.	.	.	.	.
*4B1-l (*LYQE*)	.	.	.	.	C	.	G	.	.	.	G	------	.	.	.	.	.	.	T	.	.	T	.	.	C	.	.	.
*4C1-h	.	.	.	.	C	T	G	.	.	.	G	------	.	.	.	.	.	.	T	.	.	T	.	.	.	.	.	.
*4D1-h	.	.	.	.	C	.	G	.	.	.	G	------	.	.	.	.	T	.	T	.	.	T	.	.	.	.	.	.
*4E1-h	.	.	.	.	C	.	G	.	.	.	G	------	A	.	.	.	.	.	T	.	.	T	.	.	.	.	.	.
*4F1-l (*LYQC*)	.	.	.	.	C	.	G	.	.	.	G	------	.	.	.	.	.	.	T	.	.	T	.	.	.	.	.	A
*4F2A-l	.	.	.	.	C	.	G	.	.	.	G	------	.	.	.	.	.	.	T	.	A	T	.	.	.	.	.	A
*4F2B-l	.	.	.	.	C	.	G	.	.	G	G	------	.	.	.	.	.	.	T	.	A	T	.	.	.	.	.	A
*4F3-l	.	.	.	.	C	.	G	.	.	.	G	------	.	.	.	.	.	T	T	.	.	T	.	.	.	.	.	A

The positions corresponding to the SNPs and to the deletion (position of the first deleted nucleotide) are shown in the first row (Reference sequence: Y16577). Haplotype nomenclature was given according to others [17]. The common nomenclature of formerly known SNPs and haplotypes (excluding *LYPF *and *LYQE*), is given in parentheses. *1B3, *1C2 and *1F2 were not investigated for MBL concentration, and thus did not receive the designation "l" (for low MBL levels) or "h" (for high levels). For all other haplotypes, MBL levels have been reported by us and by others [2,7,9-13]. In gray: coding region of exon 1. In SNP database: *g.259C *> *T *as rs35451939, *g.273G *> *C *as rs11003125, *g.311G *> *C *as ss107796309, *g.388G *> *A *as rs7100749, *g.396A *> *C *as rs11003124, *g.456G *> *T *as rs35615810, *g.474A *> *G *as rs7084554, *g.477C *> *T *as ss107796300, *g.478G *> *A *as ss107796301, *g.482A *> *G *as ss107796302, *g.487A *> *G *as rs36014597, *g.495delAAAGAG *as rs10556764, *g.578G *> *A *as rs35236971, *g.598C *> *A *as ss107796311, *g.602G *> *C *as rs7096206, *g.658C *> *A *as ss107796303, *g.659C *> *T *as ss107796304, *g.712A *> *T *as ss107796305, *g.753C *> *T *as rs11003123, *g.788T *> *C *as ss107796312, *g.797C *> *A *as rs45602536, *g.826C *> *T *as rs7095891, *g.925C *> *G *as ss107796306, *g.926T *> *G *as ss107796307, *g.965G *> *C *as ss107796308, *g.1045C *> *T *as rs5030737, *g.1052G *> *A *as rs1800450, *g.1061G *> *A *as rs1800451.

We identified in this and in other studies 14 haplotypes belonging to clade *1 and 9 haplotypes belonging to clade *4 and added data from others for comparison (Table 2). Eight of the first 14 and 6 of the last 9 haplotypes were polymorph in at least one population. Among the rare haplotypes, we found three previously unknown in the European population: *1B3, a rare *HYPA-*similar haplotype; *1C2, the only *LXPA-*similar haplotype; and *4C1-h, a *LYQA*-similar haplotype with a *g.456G *>*T *SNP found in three heterozygotes (the first two haplotypes were singletons). The *g.456G *>*T *SNP was assigned by others to an otherwise *HYPA *haplotype reconstructed from unphased genotypic data of one Sardinian heterozygote [19]. Maximum likelihood phasing of our own data with the EM and ELB algorithms generated 1-2% erroneously assembled haplotypes in the Gabonese and European samples. Only in the Gabonese, seven spurious "new" haplotypes were generated with the EM and eleven with the ELB algorithm (Table 3). To verify the effect of sample size in frequency estimates, we compared the haplotype distribution between some populations investigated by us and by others [19]. Although there were no significant differences with the exact population differentiation test, differences between individual haplotype frequencies were significant, even between samples with similar ancestry (Table 2).

**Table 2:** *MBL2 *haplotype frequencies (%) in diverse populations.

	***Afro-Gabonese ***^**(1)**^	**Afro-Americans **^**2**^	***German Europeans ***^**(1)**^	**Euro-Americans **^**2**^	***Euro-Brazilians ***^**(1)**^
Haplotypes	N = 856 (N = 64)	N = 48	N = 208 (N = 48)	N = 62	N = 262
*1A1-h (*LYPA*)	16.8 (9.5)	27.1	4.81 (8.3)	1.61	3.44
*1A1.1B2-l (*LYPD*)	0	0	0	0	0.38
*1B1-h (*HYPA*)	5.37 (6.3)	6.25	29.8 (41.7)	35.5	29.39
*1B2-l (*HYPD*)	0.12 (0)	2.08	8.17 (10.4)	9.68	6.49
*1B3	0	0	0	0	0.38
*1C1-l (*LXPA*)	14.6 (18.8)	14.6	21.6 (8.3) *	16.1	20.23
*1C2	0	0	0.48	0	0
*1D1-h	3.09 (0)	0	0	0	0
*1E1-h (*LYPF*)	0.12 (0)	0	0	0	0
*1F1-l (*LYPB*)	2.45 (1.6)	2.08	11.1 (10.4)	12.9	14.89
*1F2 ¶	0	0	0	0	0
*1G1-h	0.12 (0)	0	0	0	0
*1H1-h	7.01 (3.1)	6.25	0	0	1.15
*1H2-h	0.12 (0)	0	0	0	0.38
*1J1-h	0.82 (3.1)	0	0	0	0
*2A1-h	0.7 (0)	0	0	0	0
*3A1-h	0.12 (0)	0	0	0	0
*4A1-h (*LYQA*)	25.6 (40.6) **	18.8	23.1 (20.9)	24.2	20.99
*4B1-l (*LYQE*)	0.23 (0)	0	0	0	0
*4C1-h	0	0	0.48 (0)	0	0.76
*4D1-h	0.23 (1.6)	0	0	0	0
*4E1-h	5.37 (1.6)	6.25	0	0	0
*4F1-l (*LYQC*)	16.7 (12.5)	14.6	0	0	0.76
*4F2A-l	1.75 (1.6)	0	0.48 (0)	0	0.76
*4F2B-l	0.58 (0)	0	0	0	0
*4F3-l	1.17 (0)	2.08	0	0	0

	**North Chinese ^3 (1)^**	**Hispanics ^2^**	**Pacific Rim ^2^**	**Guarani ^4^**	**Kaingang ^4 (1)^**
**Haplotypes**	**N = 348 (N = 48)**	**N = 46**	**N = 48**	**N = 158**	**N = 126 (N = 26)**

*1A1-h (*LYPA*)	0	6.52	2.08	0.63	1.59 (0)
*1A1.1B2-l (*LYPD*)	0	0	0	0	0
*1B1-h (*HYPA*)	54.9 (31.3) **	28.3	45.8	48.1	52.4 (65.4)
*1B2-l (*HYPD*)	0	4.35	0	0	0
*1B3	0	0	0	0	0
*1C1-l (*LXPA*)	14.1 (22.9)	19.6	10.4	0	0.79 (0)
*1C2	0	0	0	0	0
*1D1-h	0	0	0	0	0
*1E1-h (*LYPF*)	0	0	0	0	0
*1F1-l (*LYPB*)	14.1 (29.2)^&^**	13.0	16.7	47.5	26.2 (23.1)
*1F2	0	0	0	0	1.59 (0)
*1G1-h	0	0	0	0	0
*1H1-h	2.87 (4.2)	2.17	16.7	0	15.9 (11.5)
*1H2-h	0	0	0	0	0
*1J1-h	0	0	0	0	0
*2A1-h	0	0	0	0	0
*3A1-h	0	0	0	0	0
*4A1-h (*LYQA*)	14.1 (6.3)	19.6	8.33	3.8	0.79 (0)
*4B1-l (*LYQE*)	0	0	0	0	0
*4C1-h	0	0	0	0	0
*4D1-h	0	0	0	0	0
*4E1-h	0	2.17	0	0	0
*4F1-l (*LYQC*)	0	2.17	0	0	0
*4F2A-l	0	2.17	0	0	0
*4F2B-l	0	0	0	0	0
*4F3-l	0	0	0	0	0.79 (0)

N number of chromosomes (N = 162 for *1D1-h *in Afro-Gabonese, and this is why the sum of frequency values in this group did not equal exactly 100%). The distribution of *MBL2 *genotypes in the Gabonese adult sample as a whole was homogeneous with the distribution that we formerly found in a sample of 284 Gabonese children (39 malaria-free, 136 with uncomplicated malaria and 109 with severe malaria but not with cerebral malaria and/or severe hypoglycemia) [2]. In parentheses: differing values found in samples of smaller size but similar ethnic background (Gabonese Bantu, Danish, Chinese and Colombian Piapoco, respectively) [19]. & the Chinese investigated by others presented an additional *LYPB *haplotype with 6.3% frequency and a *g.634G *> *A *SNP (called by them *-258 G *> *A*) [19]. In italics: this work; 1 [19]; 2 [18]; 3 [28]; 4 [22].* p < 0.05 ** p < 0.01

**Table 3:** Performance of haplotyping algorithms.

Population	n	Ambiguous genotypes	Expectation maximization (EM)		Pseudo-Bayesian (ELB)	
			Wrongly assembled haplotypes	Spurious "new" haplotypes	Wrongly assembled haplotypes	Spurious "new" haplotypes
Afro-Gabonese	428	59.6%	1.1%	7	2.2%	11
German Europeans	104	63.5%	1.4%	1	1.4%	2
Euro-Brazilians	131	66.4%	1.1%	2	0.8%	1
Guarani	79	61%	0	0	0	0
Kaingang	63	71%	0	0	0.8%	0

n = number of individuals.

**Figure 2:**
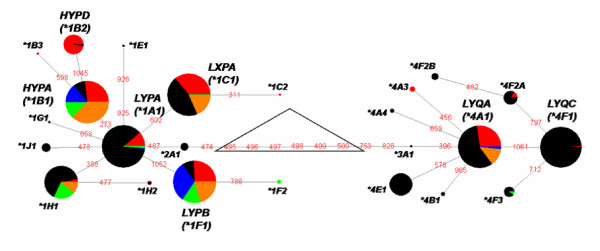
**Mutational network of *MBL2 *haplotypes**. The size of each node is proportional to the haplotype frequency in the pooled sample (this work and [18,28]). Variant nucleotide positions are indicated in red. In black: African; red: European; orange: North Chinese; blue: Guarani; green: Kaingang.

**Figure 3:**
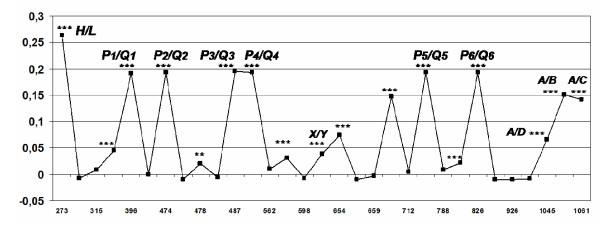
**Global Fst values distributed along the sequence**. Fsts were calculated using the data of Table 2 and of [19]. Nucleotide positions corresponding to variant sites are shown on the x-axis. *** p < 0.001, ** p < 0.01.

With the exception of *LYQA *(*4A1-h), *4 haplotypes are well represented only in the African population. In contrast, *HYPA *(*1B1-h) and *LYPB *(*1F1-l) are among the *1 haplotypes that reach high frequencies in the European, Asian and Native American, but not in the African population (Figure 2). The uneven haplotype distribution around the world is reflected by the average Fst value among all segregating sites (0.1831, *P *< 0.00001), which indicate great genetic differentiation between the analysed populations. One of the lowest individual significant Fst values corresponded to the *X/Y *SNP, whereas the highest values corresponded to the *H*, *L *and *P*, *Q *segregating sites (Figure 3). The time to the most recent common ancestor of the *MBL2 *alleles was inferred at 73,251 years ago [95% CI 5,220 - 214,440]. The mean coalescence time implies that the ancestor of groups *1 and *4 alleles were separated before the modern human dispersal from Africa [21]. The TMRCA of groups *1 and *4 was estimated to be ca. 55,000 years ago, which also indicates that the presence of alleles of African populations in both clades is a result of an ancient ancestry.

*1B-derived haplotypes, even those found using maximum-likelihood phasing by others [19], seem to be restricted to Euroasiatic populations. Beyond those described in this work, we recently identified *1B4 in the Euro-Brazilian population. This haplotype is similar to *HYPA *but with a synonymous substitution in codon 44 (also called *HYPG*) [16]. To our knowledge, *LXPA *(*1C1-l) has only one rare similar haplotype (*1C2), identified in one European individual. We also found only one *LYPB-*similar haplotype (*1F2), but others cite another four [19]. Each occur with frequencies around 2% in Asian/Amerindian groups (Ashkenazi Jewish, Japanese, Chinese and Kaingang), but three were defined by SNPs upstream to the region analysed in this study. The *1H1-h haplotype has a similar global distribution as the commonly investigated haplotypes and is well represented in African, Asian and Amerindian(-derived) populations, being less frequent in European groups. We found a similar haplotype (*1H2-h) once in a Gabonese and once in a Euro-Brazilian individual. All other clade *1 haplotypes are concentrated in African groups. *1E1-h has a rare coding mutation found only once in the Gabonese, as *1G1-h [2]. The *1D1-h haplotype, which we found with 3% frequency in this population, was found by others with comparable frequencies (1.6 - 4.2%) in the Mbuti Pygmy, Nigerian Yoruba and Somali populations [19]. *1J1-h was also found with 1.6% and 0.8% frequencies in Tanzanian Chagga and in the Somali groups, respectively. *2A1-h and *3A1-h are intermediate between *P *and *Q *containing haplotypes and most probably reminiscent of the ancient original *MBL2 *haplotype [2]. The *LYQA-*similar *4B1-l haplotype carries a coding mutation and was found only once in the Gabonese, as the *LYQC*-similar haplotype *4F2B-l. In addition to the Gabonese, *4D1-h was found by others with 1.6% frequencies in the Tanzanian Chagga [19]. *4E1-h has a SNP within a glucocorticoid responsive element and seems to be well distributed in Africa, except in the Mbuti and Baka Pygmies [19]. *4F2A-l *was formerly found associated with severe malaria [2] and has a similar distribution, except for the fact that it is also present in South-West Asian and European(-derived) groups with 11.9% (Ashkenazi Jewish [19]) to 0.5% (Germans, this work) frequencies. *4F3-l was also found in the Biaka Pygmy (2.1%), Nigerian Yoruba (1.6%) and Tanzanian Chagga (4.7%) groups [19], as well as in Afro-Americans [18] and in one individual of the Kaingang Amerindian population, known to be of mixed ancestry [22].

**Figure 4:**
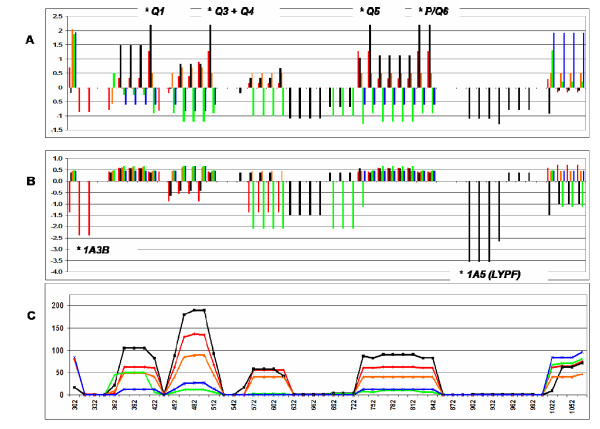
Sliding window plot of **(A) **Tajima's D values, **(B) **Fu and Li's D* values and **(C) **nucleotide diversity for the entire sequenced region. Statistics were calculated for overlapping windows of 60 bp, placed at 15 bp intervals along the sequence. * P < 0.05.

Tajima's D was significant in those regions containing five of the six *P*, *Q *segregating sites in the Gabonese population (Figure 4A). Yet Fu and Li's D* was significant in regions with rare SNPs: the *LXPA*-derived *1C2 haplotype in Europeans and the *LYPA-*derived *1E1-h haplotype in the Gabonese (also called *LYPF *due to a non-synonymous SNP in the exon 1 region) (Figure 4B). Highest nucleotide diversity was registered in the same windows with Tajima's D peaks (Figure 4C). None of the neutrality tests employed for the whole sequence or parts of it yielded significant results (Table 4).

**Table 4:** *MBL2 *sequence diversity parameters of several populations.

		***Afro-Gabonese***	**Afro-Am.**^**2**^	***European***	**Euro-Am.**^**2**^	**N. Chin.**^**3**^	**Hispanics**^**2**^	**Pac. Rim**^**2**^	**Kaingang**^**4**^	**Guarani**^**4**^
	N	856	48	470	62	348	46	48	126	158
**Promoter, exon 1 and part of intron 1**	S	22	13	16	9	9	13	9	11	7
	π	44.1	43.7	37.6	37.6	27.8	40.4	25.2	17.4	16.9
	θ_W_	37.0	36.1	29.3	23.6	17,3	36.5	25.0	27.4	15.3
	D_T_	0.459	0.63	0.672	1.591	1.297	0.325	0.024	-0.934	0.23
	D_F_	-1.157	-0.004	-0.404	1.343	1.23	0.012	1.353	-0.498	1.159
	F	-0.583	0.245	0.028	1.68*	1.51	0.137	1.085	-0.782	0.996
	H	-1.28	0.743	-0.276	-0.387	-1.718	0.039	-2.163#	-3.27*	-3.282*
**5' upstream regulatory region**	S	16	10	13	7	8	10	8	9	6
	π	52.1	51.8	43.4	43.1	32.8	46.9	28.7	1.62	1.42
	θ_W_	35.6	36.8	31.5	24.3	20.3	37.1	29.4	30.2	17.4
	D_T_	1.044	1.177	0.858	1.963	1.268	0.762	-0.063	-1.146	-0.383
	D_F_	0.129	0.791	-0.783	1.227	1.166	-0.406	1.302	-0.1	1.083
	F	0.606	1.08	-0.181	1.73*	1.446	-0.03	1.015	-0.56	0.693
	H	-1.579	0.434	-0.619	-0.74	-1.921#	-0.32	-2.39#	-3.534*	-3.33*
**Exon 1 coding region**	S	6	3	3	2	1	3	1	2	1
	π	19.3	19.6	19.6	21.7	13.00	21.5	15.2	22.5	26.8
	θ_W_	41.3	36.1	22.5	22.8	8.3	36.5	12.0	19.8	9.5
	D_T_	-0.92	-0.953	-0.183	-0.079	0.52	-0.866	0.352	0.202	1.925
	D_F_	-2.55*	-1.7	0.717	0.726	0.43	0.9	0.547	-1.124	0.466
	F	-2.386	-1.718	0.499	0.564	0.544	0.432	0.567	-0.833	1.071
	H	0.30	0.308	0.343	0.353	0.203	0.359	0.227	0.265	0.048

Am. American European include the German Europeans and Euro-Brazilians, which were homogeneous at the genotypic level N. Chin. North Chinese Pac. Pacific; N number of chromosomes; S number of segregating sites; π (×10^-4^) nucleotide diversity per site, θ_W _(×10^-4^) Watterson's Theta per site from S; D_T _Tajma's D; D_F _Fu and Li's D without an outgroup (D*); F Fu and Li's F without an outgroup (F*); H Fay and Wu's H with the chimpanzee (Genbank: AY970685.1) as an outgroup. In italics: this work; 2 [18]; 3 [28]; 4 [22]. * P < 0.05 # P < 0.10

## Discussion

Both circulating levels of MBL oligomers and functional activity have been correlated with common *MBL2 *genetic variants. There are at least 28 segregating sites in the *MBL2 *promoter and exon 1 sequence [23], and 26 allelic haplotypes were physically defined in this study. Nucleotide diversity in Afro-derived populations reached 5 × the average value of chromosome 10 (8.25 × 10^-4^) [24], where the *MBL2 *gene resides (10q11.2→q21). This is still 2 × less than the lowest values found for polymorphic MHC regions (1%) [25], indicating that the *MBL2 *promoter-exon1 nucleotide diversity is intermediate among immune protein coding genes.

Several of the newly identified haplotypes are polymorph and of interest for disease association studies. Nevertheless beside the *A/B/C/D *system adopted for exon 1 alleles since 1991 [26] and of the *H*, *L*, *X*, *Y *and *P*, *Q *names for promoter SNPs since 1998, no other nomenclature was suggested. We adopted a phylogenetic approach that easily accommodates new haplotypes following a logical order, and suggested a way to call eventual recombinant haplotypes, incorporating knowledge about MBL serum levels.

Nevertheless haplotypes generated with EM and ELB haplotyping algorithms should be included with caution, especially when containing singletons. In our comparison, EM and ELB algorithms allowed for 1-2% errors in populations with high nucleotide diversity (π). The pseudo-Bayesian ELB performed worse in groups with very high π values, as Africans, generating more spurious "new" haplotypes. We did not find six of the haplotypes reconstructed by others using the Bayesian method implemented in PHASE software [19]. Two were recombinant (*LYQB *and *HXPA*), one presented a SNP that we haplotyped to *LYQA *and three were *LYPA*-similar haplotypes that seemed to be restricted to Pygmy populations, with SNPs presenting high Fst values. To avoid the inclusion of false haplotypes in the nomenclature system, we followed the approach of a group which only analysed haplotypes having a minimal frequency of 10% [27]. Two of the Pygmy haplotypes fulfilled this requirement, but all other haplotypes should ideally be phased by a physical haplotyping technique before inclusion.

Others used sample sizes at least four times smaller than ours [18,19]. This caused discrepant frequency results especially for the most common haplotypes. Since rare variants are not easily detected in small population samples, we also found considerable differences between our Fu and Li's D* and F* and other's results [18]. Indeed, two singletons caused significant D* values in regions with very low nucleotide diversity levels specifically in our European and Gabonese samples.

We added data from other studies [2,18,19,22,28] to calculate the Fst statistic. This approach resulted in much higher Fst values for the whole gene (0.18), than those found previously by others (0.06 [18]) and by us using only the Amerindian and Chinese samples (0.12, [22]). The same was true for the *H/L *and *P/Q *SNPs (Fst values around 0.2-0.25, compared to published 0.1-0.15, [18]), which indicate that they are good markers for population differentiation. As opposed to these high Fst values, the *X/Y *SNP presented values lower than 0.05 in this and in another study [18], compatible with global balancing selection.

**Figure 5:**
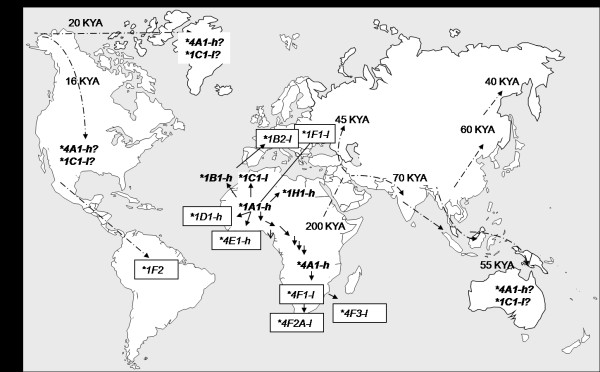
**Distribution and origins of the *MBL2 *alleles in the world**. Arrows denote the mutational steps between haplotypes (six between *1 and *4) and when dotted, the ancient migratory routes with their approximate ages [51]. The haplotypes which could have been lost by natural selection and/or genetic drift were denoted by '?'. In bold: haplotypes generated before human out-of-Africa migration. Squared: more recent haplotypes, with geographically restricted distribution. KYA thousand years ago.

We previously discussed the origin and distribution of the *LYPA *(*1A1-h), *HYPA *(*1B1-h), *HYPD *(*1B2-l), *LXPA *(*1C1-l), *LYPB *(*1F1-l), *LYPD *(*1A1.1B2-l), *LYQA *(*4A1-h) and *LYQC *(*4F1-l) haplotypes [22]. In general, the most frequent clade *1 haplotypes are globally distributed, whereas clade *4 haplotypes are more restricted to the African continent. Four of the five most ancient haplotypes also belong to clade *1: *1A1-h, *1B1-h, *1C1-l and *1H1-h. Among them, only *1C1-l (with the *X *variant) is associated with low (although complement-activating) MBL production. This and the *4A1-h haplotype do not naturally occur in native Aboriginal, Greenlandic and Amerindian populations [11,22,29,30], having probably been lost through bottleneck effects along the migration routes. The other eight polymorph haplotypes (with a frequency higher than 1%) have probably had a more recent origin, being geographically more restricted. Among them, only two are associated with high MBL levels: *1D1-h and *4E1-h. All others generate low MBL levels that, in addition, are greatly restricted in complement activation due to the *B*, *C *and *D *mutations, which occur in critical residues of the collagen-like region (*1B2-l, *1F1-l, *4F1-l, *4F2A-l, *4F3-l and *1F2) (Figure 5). Interestingly, the *MBL1P1 *pseudogene has been selectively turned off during evolution through the same molecular mechanisms causing the non-functional recent *MBL2 *haplotypes in man [31]. A more restricted distribution is obviously the case of all haplotypes containing singletons, as well as of *1J1-h, *4D1-h and *4F3-l in Africa, *1A1.1B2-l, *1B2-l and *4C1-h in Europe. They are therefore characteristic of different ethnic groups.

The clades *1 and *4 are separated by six mutational steps (*P*, *Q *variants), which probably occurred before the human out-of-Africa migration (Figure 5). Of these six segregating sites, probably the most ancient is the *g.487G *>*A *variant and the most recent, the *g.396A *>*C *variant [2]. *Q *variants are less widely distributed than *P *variants, justifying their high Fst values. They are functionally associated with higher promoter activity [6,32] and five of them presented positive, significant Tajima's D values in the Gabonese population. A significant positive value for Tajima's D test indicates an excess of intermediate-frequency variants, as compared with expected frequencies under neutrality, and constitutes evidence of balancing selection (mutations leading to higher MBL levels could have been selectively retained in the ancient human population) or population subdivision. Nevertheless the emergence of several recent mutations as well as genetic drift erased the selective signature at the long haplotype scale, leading to non-significant, although positive, Tajima's D values for the whole haplotype in this and in other studies (eg. Table 4), one of which included 1,166 chromosomes from 24 worldwide populations [18,19,22]. The patterns of *MBL2 *variation at the large temporal scale would thus have been shaped by stochastic evolutionary factors and therefore be compatible with neutral evolution.

## Conclusions

In this work, we evaluated the *MBL2 *promoter-exon 1 region using haplotype-specific sequencing in more than 700 chromosomes and found three new European haplotypes. We propose a phylogenetic nomenclature to standardize *MBL2 *studies and found two major phylogenetic branches due to six strongly linked polymorphisms associated with high MBL production. They present high Fst values and are imbedded in regions with high nucleotide diversity and significant Tajima's D values. Compared to others using small sample sizes and unphased genotypic data, we found differences in haplotyping, frequency estimation, Fu and Li's D* and Fst results. Using extensive testing for selective neutrality, we confirmed that stochastic evolutionary factors have had a major role in shaping this polymorphic gene worldwide.

## Methods

### Subjects and samples

We investigated 104 German Europeans, 131 Euro-Brazilians and 144 Gabonese adults. The German Europeans were healthy unrelated students and employees of the University of Tübingen, enrolled as controls in a genetic association study with type 2 diabetes, approved by the Ethics Committee of the University of Tübingen in Germany [33]. The Euro-Brazilians were healthy blood donors with mixed, however predominantly European ancestry, resident in Paraná state, South Brazil, sampled for different association studies, all approved by the Ethics Committee of Research in Humans of the Clinical Hospital, Federal University of Paraná, Brazil [16,34,35]. The Gabonese individuals took part in a large epidemiologic survey to detect the prevalence of asymptomatic *Plasmodium falciparum *infection in the villages around Lambaréné, Gabon, a study approved by the ethics committee of the International Foundation Albert Schweitzer Hospital [36]. All individuals signed an informed consent form prior to their inclusion in these studies.

### MBL2 typing

DNA was collected with anticoagulant ethylenediaminetetraacetic acid and extracted from peripheral blood mononuclear cells through standard salting-out and phenol/chloroform/isoamyl alcohol methods. A fragment of 1059 nucleotides was amplified using the forward primers MBLfor (5'-ATGGGGCTAGGCTGCTGAG-3') and the reverse primer MBLrev (5'-CCAACACGTACCTGGTTCCC-3'). Sequence specific (SSP) PCR products were generated using the same reverse primer, combined to forward primers specific for variant *H *(Hf: 5'-GCTTACCCAGGCAAGCCTGTG-3') or for the variant *L *(Lf: 5'-GCTTACCCAGGCAAGCCTGTC-3'); for the variant *X *(Xf: 5'-CCATTTGTTCTCACTGCCACC-3') or for the variant *Y *(Yf: 5'-CCATTTGTTCTCACTGCCACG-3'). The PCR products with the primers Hf or Lf with MBLrev and Xf or Yf with MBLrev were 837 and 508 nucleotides in length, respectively. Hf and Lf were also combined to specific reverse primers for the variant *P *(Pr: 5'-CTCAGTTAATGAACACATATTTACCG-3') or for the variant *Q *(Qr: 5'-CTCAGTTAATGAACACATATTTACCA-3'), generating a product of 599 nucleotides. All fragments were sequenced with the amplification primers or with an internal exon 1 sequencing primer, MBLint (5'-GAGGCCAGGGATGGGTCATC-3'), using Big dye terminator version 1.1 chemistry (Applied Biosystems, Foster City, CA). Amplification conditions are described in detail elsewhere [20]. The reactions were purified with the Performa DTR V3 system (Edge BioSystems, Gaithersburg, MD) and analyzed on an automated sequencer (ABI Prism 3100 Genetic Analyzer, Applied Biosystems, Foster City, CA). New variants (singletons) were verified by reamplification and resequencing.

### Statistical analyses

Genotype and haplotype frequencies were obtained by direct counting. We tested for deviations from Hardy-Weinberg proportions with the exact test of Guo and Thompson [37]. The haplotype frequency distributions of the populations examined by our group and by others were compared by applying the exact test of population differentiation of Raymond and Rousset [38]. Genetic differentiation among populations was estimated from haplotype frequencies using the Fst statistic, based on the analysis of molecular variance [39]. To verify the effect of other methods to infer haplotypes compared to physical haplotyping of SNPs, we simulated our own data using the (maximum-likelihood) EM algorithm or the (pseudo-Bayesian) ELB algorithm, with the settings recommended by the authors [40,41]. These statistical analyses were done using the software package ARLEQUIN version 3.1 [42]. Fisher's exact tests were performed for differences between individual haplotype frequencies, using SISA software package http://home.clara.net/sisa.

We calculated the following summary statistics of genetic diversity: the number of polymorphic sites (S), the nucleotide diversity over loci (π) and Watterson's θ, defined as 4Neμ, where Ne is the effective population size and μ, the estimated mutation rate. We examined deviation from neutrality-equilibrium conditions using Tajima's D statistic [43], Fu and Li's D and Fu and Li's F without an outgroup (also known as D* and F*) [44] and Fay and Wu's H [45] tests. Significance was assessed by comparing the observed values to 10^4 ^coalescent simulations, conditional on the observed sample size and on the value of S or on the value of θ, assuming a standard neutral model with no recombination. Deletions were excluded from all analyses. To see if deviation from selective neutrality can be found in specific regions of the gene, we also tested the 5' upstream regulatory region (which includes the non-coding *P*, *Q *SNP) and the exon 1 coding region separately. The heterogeneity in π values and Tajima's D statistic across the sequenced region was also examined by use of the sliding window feature of the DnaSP program. Statistics were calculated for overlapping windows of 60 bp, placed at 15 bp intervals along the sequence. Neutrality tests and sequence diversity parameters were calculated using the DnaSP version 4.10.1 software [46].

The Network 4.1.1.2 package http://www.fluxus-technology.com/sharenet.htm was used to construct the minimum-mutation network, which reflects the mutational relationships among the *MBL2 *haplotypes by means of the Median Joining (MJ) algorithm [47]. The MEGA 3.1 program was used to construct the phylogenetic maximum parsimony tree with bootstrap test http://www.megasoftware.net/. The time to the most recent common ancestor (TMRCA) of *MBL2 *was estimated using a relaxed molecular clock approach [48]. Evolutionary rate was modeled by the uncorrelated lognormal distribution and a coalescent prior (Bayesian skyline) was assigned to the tree. The average rate of molecular evolution of the *MBL2 *gene (1 × 10^-7^) was obtained using a theta per site value of 0.0039 calculated for human sequences in DnaSP [46] and the estimate of human effective population size of 10,000 [49]. A normal prior with mean 1 × 10^-7 ^and standard deviation of 1 × 10^-7 ^was used for the rate of evolution. Divergence time inference was conducted in BEAST 1.4.8 [50]. In order to obtain the posterior distribution of divergence times, the Markov chain was sampled 50,000 times and 10% of the states were discarded as burn-in.

## Authors' contributions

ABWB carried out the molecular biological studies and wrote the manuscript. IJM-R participated in the molecular work and conducted the recruitment of subjects. ABWB, DM, CGS, MLP-E and KD participated in statistical analyses. FL, BL, and PGK conducted the recruitment of individuals for the study. JFJK supervised the molecular work and finalised the manuscript.

All authors read and approved the final manuscript.
